# The Nucleotide Excision Repair Pathway Limits L1 Retrotransposition

**DOI:** 10.1534/genetics.116.188680

**Published:** 2016-11-14

**Authors:** Geraldine Servant, Vincent A. Streva, Rebecca S. Derbes, Madushani I. Wijetunge, Marc Neeland, Travis B. White, Victoria P. Belancio, Astrid M. Roy-Engel, Prescott L. Deininger

**Affiliations:** * Department of Epidemiology, School of Public Health and Tropical Medicine, Tulane Cancer Center, Tulane University, New Orleans, Louisiana 70112; † Department of Structural and Cellular Biology, School of Medicine, Tulane Cancer Center, Tulane University, New Orleans, Louisiana 70112; ‡ Tulane Center for Aging, Tulane University, New Orleans, Louisiana 70112

**Keywords:** L1 retrotransposon, nucleotide excision repair, target-primed reverse transcription, DNA damage, genome stability

## Abstract

Long interspersed elements 1 (L1) are active mobile elements that constitute almost 17% of the human genome. They amplify through a “copy-and-paste” mechanism termed retrotransposition, and *de novo* insertions related to these elements have been reported to cause 0.2% of genetic diseases. Our previous data demonstrated that the endonuclease complex ERCC1-XPF, which cleaves a 3′ DNA flap structure, limits L1 retrotransposition. Although the ERCC1-XPF endonuclease participates in several different DNA repair pathways, such as single-strand annealing, or in telomere maintenance, its recruitment to DNA lesions is best characterized in the nucleotide excision repair (NER) pathway. To determine if the NER pathway prevents the insertion of retroelements in the genome, we monitored the retrotransposition efficiencies of engineered L1 elements in NER-deficient cells and in their complemented versions. Core proteins of the NER pathway, XPD and XPA, and the lesion binding protein, XPC, are involved in limiting L1 retrotransposition. In addition, sequence analysis of recovered *de novo* L1 inserts and their genomic locations in NER-deficient cells demonstrated the presence of abnormally large duplications at the site of insertion, suggesting that NER proteins may also play a role in the normal L1 insertion process. Here, we propose new functions for the NER pathway in the maintenance of genome integrity: limitation of insertional mutations caused by retrotransposons and the prevention of potentially mutagenic large genomic duplications at the site of retrotransposon insertion events.

RETROTRANSPOSONS, including Long INterspersed Element 1 (L1), constitute about a third of the human genome (Lander *et al.* 2001; de Koning *et al.* 2011). D*e novo* insertions of retrotransposons have been reported as the cause of over 90 genetic diseases, indicating that these elements continue to amplify in the human genome (Ostertag and Kazazian 2001; Xing *et al.* 2007; Belancio *et al.* 2008a; Hancks and Kazazian 2012, 2016). Retrotransposons amplify throughout the genome using a “copy-and-paste” mechanism, termed retrotransposition, based on the reverse transcription of an RNA intermediate (Boeke *et al.* 1985). The L1-encoded proteins, ORF1p and ORF2p, are responsible for the amplification of L1 elements in the genome (Mathias *et al.* 1991; Feng *et al.* 1996; Moran *et al.* 1996). Reverse transcription of L1, a non-LTR (long terminal repeat) retrotransposon, occurs in the nucleus through a proposed process called target-primed reverse transcription (TPRT) (Luan *et al.* 1993; Feng *et al.* 1996; Luan and Eickbush 1996), diagrammed in Figure 1A. In the TPRT model, the L1 ORF2p-encoded endonuclease cleaves between the T and the A of a consensus sequence in the DNA (5′-TTTT/AA-3′), freeing a 3′ T-rich DNA end that primes the reverse transcription from the polyA tail of the L1 mRNA. A 3′ flap DNA structure is thought to be generated resulting from the elongation of L1 complementary DNA (cDNA) at the insertion site (Figure 1A) (Feng *et al.* 1996; Luan and Eickbush 1996; Boeke 1997; Christensen *et al.* 2006). The factors involved in the completion of the insertion process are unknown but a second nick must be made for second-strand DNA synthesis to occur.

**Figure 1:**
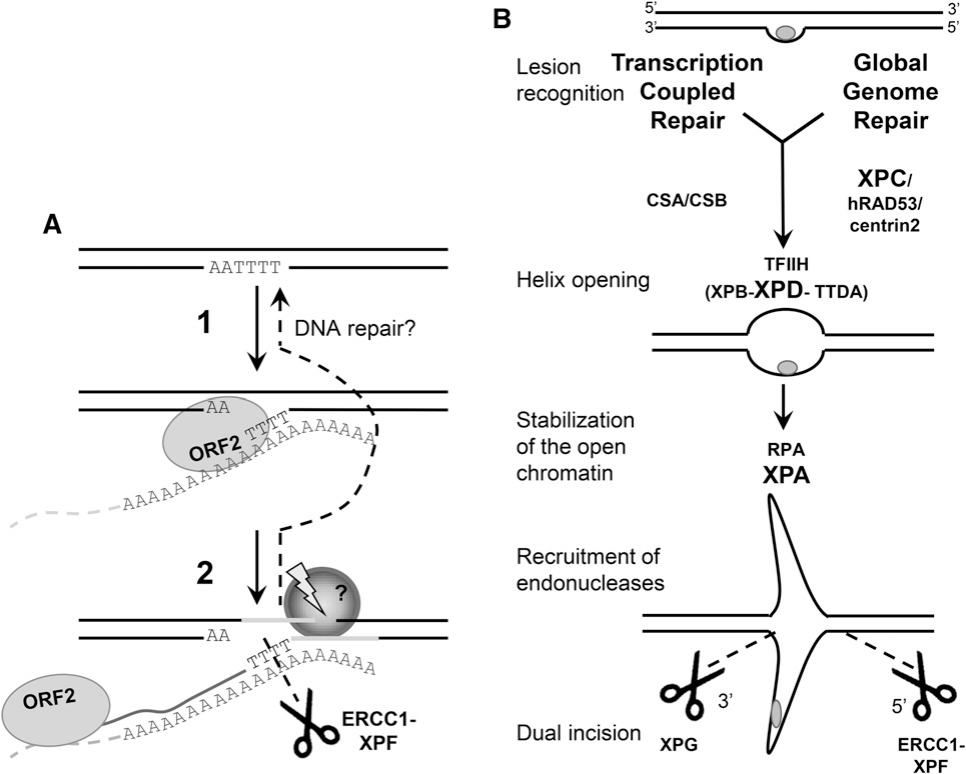
A similar 3′ flap DNA structure is generated during the L1 insertion process (TPRT) and the NER pathway. (A) Schematic of the first steps of L1-TPRT reaction. (1) ORF2 endonuclease recognizes a consensus sequence 5′-TTTTAA-3′ and cleaves the DNA between the T and A nucleotides resulting in a T-rich free 3′ end. (2) This end is allowed to base-pair with the polyA tail of the L1 mRNA (orange) and prime the reverse transcription of the mRNA. In this model, the proposed structure formed by the elongating cDNA is a 3′ flap intermediate. This 3′ flap intermediate, a known substrate for the structure-specific endonuclease ERCC1-XPF, is proposed to be cleaved, limiting L1 retrotransposition and leading to the restoration of the original DNA sequence. (B) Schematic model of the first steps of the human NER pathway. The NER pathway consists of two subpathways: TCR and GGR. Choice of pathway is determined by DNA lesion recognition. In the TCR subpathway, the base lesion in actively transcribed regions of the genome induces the arrest of transcription elongation by RNAPII. CSA and CSB proteins are recruited to the site of stalled RNAPII and initiate the repair process. In the nontranscribed genomic regions, NER repair occurs through the GGR subpathway. A wide variety of DNA base damage is detected by the DNA lesion-binding protein XPC through the structural distortion of the DNA helix. The next steps of the repair are identical in both subpathways. Once the base lesion is recognized and signaled, the general transcription factor TFIIH, a complex of 10 components including the helicases XPD and XPB, is recruited to the damage site and responsible for DNA unwinding around the lesion. XPA-RPA proteins stabilize the opened chromatin structure and recruit the endonuclease ERCC1-XPF to the damage site. XPG endonuclease is recruited with the complex TFIIH and seems to be required for the DNA unwinding. XPG incises the damaged strand at the 3′ end of the lesion and ERCC1-XPF excises 5′ of the damage. Proteins in bold are the factors evaluated in the present study. cDNA, complementary DNA; GGR, global genome repair; mRNA, messenger RNA; NER, nucleotide excision repair; RNAPII, RNA Polymerase II; TCR, transcription coupled repair; TPRT, target-primed reverse transcription.

We have previously reported that the enzymatic complex ERCC1-XPF, a 3′ flap endonuclease utilized in various DNA repair pathways, limits L1 retrotransposition (Gasior *et al.* 2008). ERCC1-XPF is a structure-specific endonuclease that nicks double-stranded DNA 5′ of a DNA lesion (de Laat *et al.* 1998). Therefore, we proposed that ERCC1-XPF cleaves the predicted flap structure formed by the elongating cDNA during L1 insertion (Figure 1A and Gasior *et al.* 2008). ERCC1-XPF has a global function in cellular DNA damage repair, notably in the removal of the DNA flap structures during single-strand annealing (SSA) repair of DSBs (Sargent *et al.* 2000; Ahmad *et al.* 2008; Al-Minawi *et al.* 2008). ERCC1-XPF is also a component of the telomeric TRF2 complex, involved in the protection of telomeres (Zhu *et al.* 2003). However, ERCC1-XPF function is best characterized in the cleavage of the damaged DNA strand in the nucleotide excision repair (NER) pathway, an important DNA repair system that removes a wide variety of lesions, including ultraviolet (UV) light-induced cyclobutane pyrimidine dimers (CPDs) and pyrimidine-(6,4)-pyrimidone photoproducts (6–4 PPs), as well as bulky chemical DNA adducts and intrastrand crosslinks (Hoeijmakers 2001; Reardon and Sancar 2005; Sugasawa *et al.* 2009). Therefore, different pathways could be involved in the recruitment of the ERCC1-XPF complex to limit L1 retrotransposition. Although a DNA flap structure has not been identified as a lesion recognized by the NER pathway, the NER lesion binding protein XPCp can recognize a flap structure *in vitro* (Sugasawa *et al.* 2001, 2002). In the present study, we thus investigated the role of the NER pathway in the regulation of L1 retrotransposition.

Rather than recognizing specific base modifications, NER senses structural DNA distortion and nonhydrogen-bonded bases, caused by DNA lesions (Sugasawa *et al.* 2009; Clement *et al.* 2010). In humans, defects in NER are associated with several autosomal recessive disorders, such as xeroderma pigmentosum (XP) and Cockayne syndrome (Lehmann *et al.* 2011; Laugel 2013). Patients suffering from these diseases exhibit an extreme sensitivity to sun exposure, neurological disorders, and a predisposition to cancers. Complementation studies have revealed nine factors (XPA through XPG, CSA, and CSB) involved in NER activity (for review, see Cleaver *et al.* 2009; Nouspikel 2009). NER consists of two subpathways that have two distinct mechanisms of lesion recognition but share a common central repair pathway: global genome repair (GGR) and transcription-coupled repair (TCR) (Figure 1B) (Vermeulen and Fousteri 2013; Puumalainen *et al.* 2016). TCR only repairs DNA lesions in the transcribed strand of active genes (Mellon *et al.* 1987). In TCR, the CSA and CSB proteins are recruited to the site of a stalled RNA Polymerase II (RNAPII) to initiate the repair (Troelstra *et al.* 1992; Henning *et al.* 1995; Groisman *et al.* 2003; van den Boom *et al.* 2004). GGR is the most active branch that detects base lesions in the remainder of the genome through the XPC complex, consisting of the XPC protein, as well as hRAD53 and centrin2 proteins (Sugasawa *et al.* 1998; Riedl *et al.* 2003). After the recognition step, both pathways converge to use a common set of proteins involved in the removal of the damaged strand (Schärer 2011). Two structure-specific endonucleases, the ERCC1-XPF complex and XPG, excise the damaged strand on the 5′ and 3′ sides of the lesion, respectively, followed by DNA replication and ligation to fill the gap (O’Donovan *et al.* 1994; Sijbers *et al.* 1996).

Our data show that multiple proteins of the GGR pathway are required to limit the mobility of the non-LTR retrotransposon L1. Additionally, new L1 inserts, recovered in NER-deficient cells, show abnormally large duplications, suggesting a potential role of NER proteins in the L1 insertion process. Therefore, we hypothesize that the GGR pathway can recognize the elongating L1 cDNA in a TPRT model of retrotransposition and excise it, inhibiting the damage caused by *de novo* L1 inserts.

## Materials and Methods

### Cell lines and culture conditions

HeLa cells (American Type Culture Collection, Rockville, MD: CCL2) were grown in Eagle’s Minimal Essential Medium (EMEM) supplemented with 10% Fetal Bovine Serum, 0.1 mM nonessential amino acids (Life Technologies), and 1 mM sodium pyruvate (Life Technologies) at 37° in a 5% carbon dioxide environment. The following cell lines were obtained from the Coriell Cells Repository: XPC-SV40-transformed fibroblast (GM15983), XPD-SV40-transformed fibroblast (GM08207), the stably complemented version of XPD-cell line (XPD+) (GM15877) and the isogenic pair of XPA-SV40-transformed fibroblasts (GM04312), and the stably complemented version (XPA+) (GM15876). XPC-, XPD-, and XPA- cell lines were grown in EMEM supplemented with 10% Fetal Bovine Serum and 0.1 mM nonessential amino acids (Life Technologies) at 37° in a 5% carbon dioxide environment. The XPD+ cell line was grown in the same conditions with the presence of 500 μg/ml G418 (Life Technologies). The XPA+ cell line was grown in Dulbecco’s Modified Eagle Medium supplemented with 10% Fetal Bovine Serum (Life Technologies).

### Plasmids

TAM102/L1.3 contains the cytomegalovirus (CMV) promoter upstream of the L1.3 element, deleted for L1 5′UTR, and the *mblastI* indicator cassette cloned in pCEP4 (Morrish *et al.* 2002).

TAMD702A/L1.3 derives from TAM102/L1.3 and contains the reverse transcriptase-deficient mutant of the L1.3 element and the *mblastI* indicator cassette cloned in pCEP4 (Morrish *et al.* 2002).

TAMH230A/L1.3 derives from TAM102/L1.3 and contains the endonuclease-deficient mutant of the L1.3 element and the *mblastI* indicator cassette cloned in pCEP4 (Morrish *et al.* 2002).

The synL1_neo vector used for the recovery of *de novo* L1 inserts was previously described (Gasior *et al.* 2007).

The pBS-L1PA1_CH__blast rescue vector is a pBS-L1PA1_CH_ plasmid (Wagstaff *et al.* 2011) tagged with a Blast rescue cassette for the recovery of *de novo* L1 inserts. (Addgene 69611)

pCMV6-XPC (ORIGENE) consists of XPC cDNA driven by the CMV promoter.

pCMV6-XPCΔ, control vector, was constructed by deleting 342 bp, between *Bgl*II (nt 753) and *Bam*HI (nt 1095) sites, in the XPC cDNA to create a defective deletion product.

pIRES2-EGFP (Clontech) contains a neomycin resistance gene expressed from a SV40 promoter that allows for the selection of transfected cells, which is used as a toxicity control. The vector also contains a multi-cloning site downstream of a CMV promoter and upstream of an IRES and eGFP marker.

pCMV-Bsd (Life Technologies) contains a blasticidin resistance gene.

Both plasmids above were used in parallel in the retrotransposition assays as a combined control for transfection and growth variations between the cell lines used.

All plasmid DNA was purified by alkaline lysis and twice purified by cesium chloride buoyant density centrifugation. DNA quality was also evaluated by the visual assessment of ethidium bromide-stained agarose gel electrophoresed aliquots.

### Retrotransposition assays

For cells with lower transfection efficiencies such as XPA-, XPA+, XPD-, XPD+, and XPC- fibroblasts, L1 retrotransposition assays were performed as described in Moran *et al.* (1996) using the L1 episomal vectors. Briefly, 5 × 10^6^ cells were seeded in T75 flasks. Cells were transfected the next day at about 90% confluence using Lipofectamine 2000 (Life Technologies) following the manufacturer’s protocol. For L1 retrotransposition assays, cells were transfected with 1 or 3 μg of L1.3 or L1.3-RT (-) construct tagged with the *mblast* retrotransposition cassette (TAM102/L1.3 or TAMD702A/L1.3) in T75 flasks. Next, 3 µg of XPC expression vector were used to transiently complement XPC-deficient cells. Cells were selected for the presence of the L1 plasmid in selective medium containing 200 µg/ml hygromycin (Life Technologies) for 5 days. The selection was removed and cells were grown for 7 days in growth medium. After the growing period, hygro^R^ cells were trypsinized and reseeded in six-well plates in triplicate at serial dilutions, from 10^6^ to 10^3^ cells, and selected for transposition events in medium containing 10 µg/ml blasticidin (InvivoGen). After 15 days, cells were fixed and stained with crystal violet solution (0.2% crystal violet in 5% acetic acid and 2.5% isopropanol). The number of blast^R^ or neo^R^ colonies was counted in each well and the relative number of colonies per 10^6^ transfected cells was determined.

### L1 toxicity and colony formation assay

L1 toxicity and colony formation assays were performed using the L1 episomal vectors. Briefly, 5 × 10^6^ cells were seeded in T75 flasks. Cells were transfected the next day at about 90% confluence using Lipofectamine 2000 (Life Technologies) following the manufacturer’s protocol. Cells were transfected with 3 μg of L1.3, L1.3-RT (-), or L1.3-EN (-) construct tagged with the *mblast* retrotransposition cassette (TAM102/L1.3, TAMD702A/L1.3, or TAMH230A/L1.3). To transiently complement XPC-deficient cells, 3 µg of XPC expression vector were used. Cells were selected for the presence of the L1 plasmid in selective medium containing 200 µg/ml hygromycin (Life Technologies) for 14 days. The cells were then fixed and stained with crystal violet solution (0.2% crystal violet in 5% acetic acid and 2.5% isopropanol). The number of hygro^R^ colonies were counted in each flask.

### Quantitative RT-PCR (RT-qPCR)

First, 5 × 10^6^ cells were seeded in T75 flasks. Cells were transfected the next day at about 90% confluence using Lipofectamine 2000 (Life Technologies) following the manufacturer’s protocol. Cells were transfected with 3 μg of L1.3 construct tagged with the *mblast* retrotransposition cassette (TAM102/L1.3). To allow for the expression of the vector, total RNA was extracted the next day from each flask using TRIzol Reagent (Life Technologies). We then carried out chloroform extraction and isopropanol precipitation. RNA was suspended in 80 µl DEPC-treated water. The cDNA was synthesized using the Reverse Transcription System (Promega, Madison, WI), following the manufacturer’s protocol. Briefly, 1 µg total RNA was denatured at 75° for 5 min. Reverse transcription reaction was primed with Oligo(dT)_15_ primers and incubated at 42° for 1 hr in a thermocycler (Bio-Rad, Hercules, CA: C1000 Touch). The enzyme was then heat-inactivated at 99° for 5 min. To avoid contamination with L1 DNA from the genome or the expression vector, and to make sure that only the L1 transcript was quantified, we analyzed the spliced *mblast* reporter cassette in L1 RNA using the TAQMAN qPCR approach. The PCR reaction (SsoFast Probes Supermix, Bio-Rad) was performed in a qPCR thermocycler (Bio-Rad, CFX96) using 1 µl of cDNA and the blasticidin primers at 250 mM final concentration each: Primer *F* (5′-GCAGATCGAGAAGCACCTGT-3′), Primer *R* (5′-TGGTGTCAATGTATATCATTTTACTGG-3′) and the 5′-FAM-labeled, ZEN internal quencher, Iowa-Black Fluorescent Quencher (IDT) probe at 150 mM final concentration (5′−/56-FAM/AGGTTGCCA/ZEN/GCTGCCGCA/3IABkFQ/-3′) as follows: initial denaturation at 95°, 45 cycles of 95° for 10 sec, 60° for 20 sec. The probe was designed to span the splice junction of the mature RNA so that it would not anneal to the unspliced DNA. The fluorescence was read during the annealing/elongation step (60°; 20 sec). For normalization, predeveloped TaqMan Assay Reagents for Human β-actin (cat# 4326315E, Life Technologies) were used. Reactions were done in triplicate. The values of the Cq for each PCR reaction were determined using Bio-Rad CFX Manager software (Bio-Rad).

### Recovery of *de novo* L1 inserts

*De novo* L1 insert recovery was performed as previously described with slight modifications, as noted below (Morrish *et al.* 2002; El-Sawy *et al.* 2005). Briefly, 3 × 10^6^ XPA-, XPD-, XPC-, or XPD+ cells were transfected with 3 µg synL1_neo rescue vector (Gasior *et al.* 2007) or pBS-L1PA1_CH__blast rescue vector using Lipofectamine 2000 reagent (Life Technologies). Cells were selected with 200 µg/ml hygromycin for 5 days, and then put under 400 µg/ml of G418 or 10 µg/ml of Blasticidin for 14 days to allow for colony formation. Neo^R^ and blast^R^ cells were harvested by trypsinization and genomic DNA was extracted using a QIAGEN DNeasy Blood and Tissue kit (QIAGEN, Valencia, CA). Genomic DNA was digested with 100 U *Hin*dIII (NEB) overnight at 37°. The following day, digested genomic DNA was self-ligated using 1200 U T4 DNA ligase (NEB) in a volume of 1 ml overnight at room temperature. DNA was purified and concentrated using centrifugal filters (Amicon Ultra, 0.5 ml, 50K, Millipore). Purified DNA was transformed by electroporation into competent DH5α *Escherichia coli* (Life Technologies). Individual kanamycin- or blasticidin-resistant colonies were grown and plasmid DNA was harvested using a SV Wizard miniprep kit (Promega). The 5′ end of the *de novo* L1 insert was sequenced using primers specific to the L1 rescue plasmid and primer walking until the 5′ end of the insert was recovered as described in Morrish *et al.* (2002). Because sequencing through a long adenosine tract at the 3′ end of the L1 inserts is not effective, the 3′ flanking genomic region was sequenced by ligation-mediated PCR based on Yuanxin *et al.* (2003) and Streva *et al.* (2015). Briefly, a pool of five to six L1 rescue vectors was digested with *Stu*I (NEB) to relax supercoils, and then sheared by sonication using a Bioruptor (Diagenode, high, 30 sec on, 90 sec off, for 12 min). Sheared plasmid DNA was primer extended using an oligo specific to the 3′ end of the L1 rescue plasmid (3′_rescue_1: 5′-ATATATGAGTAACCTGAGGC-3′ or 3′_rescue_1_secondpA: 5′-GTGGGCATTCTGTCTTGTTC-3′). Duplexed *T*-linkers were ligated using 10 U T4 DNA ligase and PCR was performed using the primers: linker-specific (5′-ACACTCTTTCCCTACACGACGCTCTTCCGATCT-3′) and 3′_rescue_1 (or 3′_rescue_1_secondpA) primers as follows: initial denaturation at 94°, 20 cycles of 94° for 30 sec, 60° for 1 min, 72° for 1 min, and a final extension for 10 min at 72°. PCR reactions were run on a 1% agarose gel and a light smear between 400 and 700 nt was gel extracted with the Qiaquick gel extraction kit (QIAGEN). Gel-extracted DNA (1 μl) was subject to an additional 15 cycles of PCR amplification as described above using linker-specific and nested 3′ rescue vector primers (3′_rescue_2: 5′-TGAGTAACCTGAGGCTATGCTG-3′ or 3′_rescue_2_secondpA: 5′-TTCTGTCTTGTTCCGGTTCTTAAT-3′) primers. The nested PCR product was run on a 1% agarose gel and the resulting smear was gel extracted and cloned into TOPO-TA (Life Technologies). Cloned PCR products were Sanger sequenced using M13 forward and reverse primers to determine 3′ end junctions. Samples were sent for sequencing to Elim Biopharmaceuticals (Hayward, CA). Lasergene 10 SeqBuilder software was utilized for sequence analysis. Flanking regions were mapped on human reference genome hg19 (build 37) using the Blat tool (https://genome.ucsc.edu/cgi-bin/hgBlat). We verified the presence of the 5′ and 3′ flanking sequences within a L1 rescue plasmid with a combination of PCR between the two regions (Supplemental Material, Table S3 for primers and Figure S8B for results). Sequences from rescues are included in File S1.

### Immunoblot analysis

To evaluate expression of NER proteins in the cells, XPA-, XPA+, XPC-, XPD-, and XPD+ cells were haverested in 300 µl of lysis buffer (50 mM Tris, pH 7.2; 150 mM NaCl; 0.5% Triton X-100; 10 mM EDTA, and 0.5% SDS). After 10 min of sonication (Bioruptor, Diagenode, manufacturer’s recommended settings), lysates were clarified by centrifugation for 15 min at 4° at 13,000 rpm and the protein concentration was determined by Bradford assay (Bio-Rad). 40 µg of proteins were loaded on 4–12% bis-tris polyacrylamide or 3–8% tris-acetate polyacrylamide gels (Life Technologies). Proteins were transferred to a nitrocellulose membrane using an iBlot gel transfer system from Life Technologies (manufacturer’s settings). The membrane was blocked for 1 hr at room temperature in PBS (pH 7.4), 0.1% Tween 20 (Sigma [Sigma Chemical], St. Louis, MO), 5% skimmed milk powder (OXOID), and then incubated overnight at 4° with an anti-XPC monoclonal antibody (D-10, sc-74410, Santa Cruz Biotechnology), anti-XPD monoclonal antibody (ab54676, Abcam), or anti-XPA polyclonal antibody (H-273, sc-853, Santa Cruz Biotechnology) diluted at 1:1000 in PBS, 0.1% Tween 20, and 3% nonfat dry milk. Membrane was then incubated for 1 hr at room temperature with the secondary donkey anti-rabbit HRP-conjugated antibody (sc-2317, Santa Cruz Biotechnology) diluted at 1:100,000 in PBS, 0.1% Tween 20, 3% nonfat milk. Signals were detected using Super Signal West Femto Chemiluminescent Substrate (Pierce Chemical, Rockford, IL).

To determine XPC protein expression, 24 hr after transfection with 3 μg of XPC expression vector or control vector, XPC-deficient cells and untransfected HeLa cells were harvested in 300 µl of lysis buffer (50 mM Tris, pH 7.2; 150 mM NaCl; 0.5% Triton X-100; 10 mM EDTA; and 0.5% SDS). After 10 min of sonication (Bioruptor, Diagenode, manufacturer’s recommended settings), lysates were clarified by centrifugation for 15 min at 4° at 13,000 rpm and the protein concentration was determined by Bradford assay (Bio-Rad). 20 µg of proteins for transfected cells and 40 µg of proteins for HeLa cells were loaded on a 3–8% tris-acetate polyacrylamide gel (Life Technologies). Proteins were transferred to a nitrocellulose membrane using an iBlot gel transfer system from Life Technologies (manufacturer’s settings). The membrane was blocked for 1 hr at room temperature in PBS (pH 7.4), 0.1% Tween 20 (Sigma Chemical), 5% skimmed milk powder (OXOID), and then incubated overnight at 4° with an anti-XPC polyclonal antibody (H-300, sc-30156, Santa Cruz Biotechnology) diluted at 1:1000 in PBS, 0.1% Tween 20, and 3% nonfat dry milk. The membrane was then incubated for 1 hr at room temperature with the secondary donkey anti-rabbit HRP-conjugated antibody (sc-2317, Santa Cruz Biotechnology) diluted at 1:100,000 in PBS, 0.1% Tween 20, and 3% nonfat milk. Signals were detected using Super Signal West Femto Chemiluminescent Substrate (Pierce Chemical).

### UV sensitivity assay

The protocol was adapted from Emmert *et al.* (2000). Briefly, 5 × 10^5^ cells were seeded in 6-cm plates and grown in growth medium for 24 hr. The growth medium was removed and the cells were irradiated in the presence of 1 ml of 1 × PBS with a bactericidal UVC lamp (254 nm, 1.57 J/m^2^/sec) at 0, 3, 6, 9, and 12 J/m^2^ UVC dose. The PBS was removed and replaced with growth medium. After 4 days, cells were counted with a hemocytometer to determine cell survival. Cell survival was calculated as the percent of live cells in the irradiated sample relative to the untreated sample. To determine the efficiency of the XPC transient complementation, 3 × 10^6^ XPC-deficient cells were transfected with 3 μg of XPC expression vector or the control vector in T75 flasks using Lipofectamine 2000 (Life Technologies) and were reseeded in 6-cm plates the next day in the conditions described above. The UV assay was then performed as mentioned above.

### Cell cycle analysis

Cells from confluent T75 plates were harvested and fixed for over 24 hr with ice-cold 70% ethanol at −20°. After fixation, cells were washed once with PBS and incubated in PI/Triton X-100 staining solution (20 μg/ml propidium iodide, 0.1% (v/v) Triton X-100, and 0.2 mg/ml RNAse A in PBS) for at least 2 hr. Flow cytometry of the stained cells was carried out on a Becton Dickinson LSRII using DiVA Software (Louisiana Cancer Research Consortium FACS Core) and 50,000 events were collected. The data were analyzed using Modfit Software (Verity Software House).

### Data availability

The authors state that all data necessary for confirming the conclusions presented in the article are represented fully within the article.

## Results

### Two essential proteins of the NER pathway, XPA and XPD, limit L1 retrotransposition

**Figure 2:**
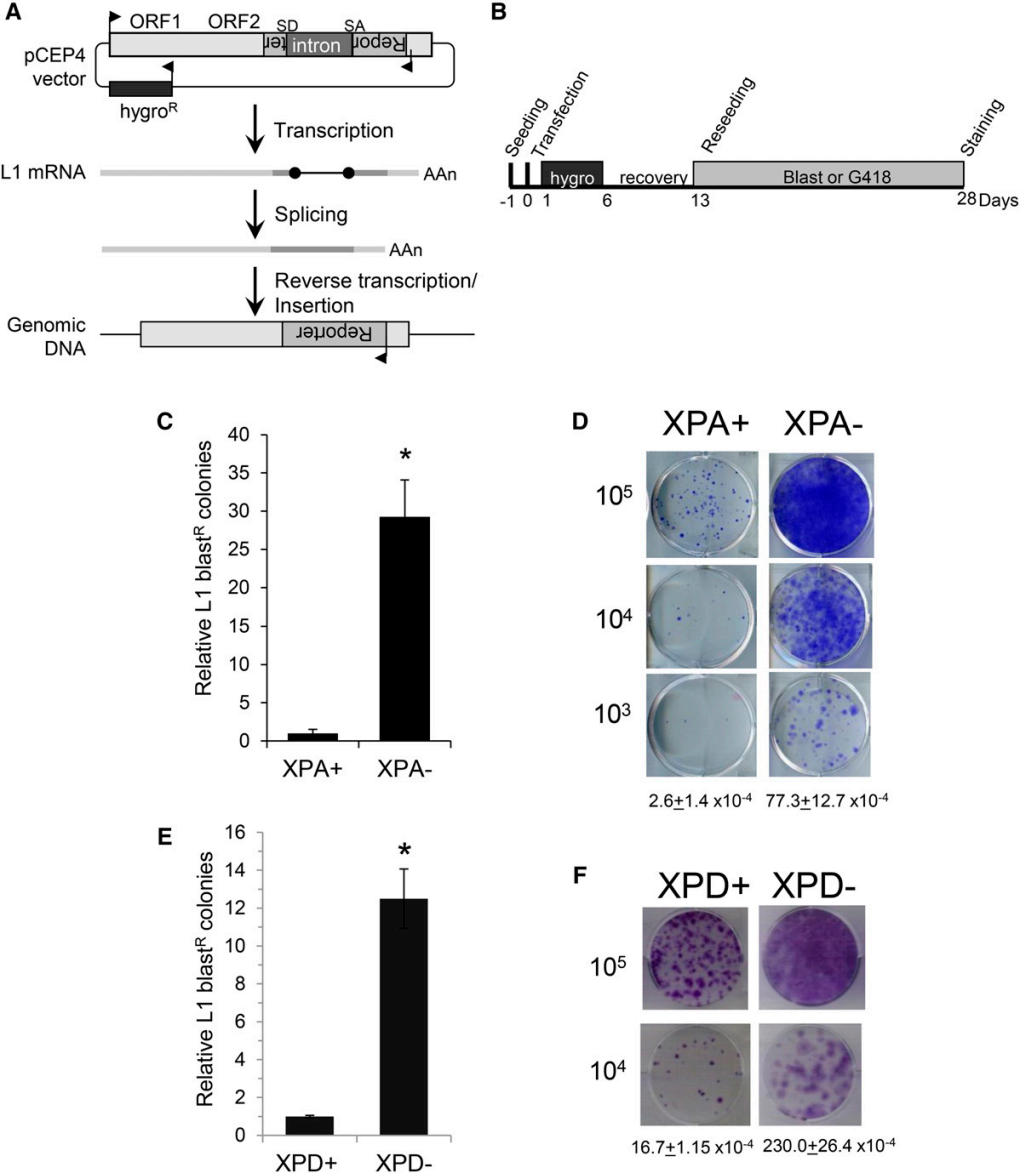
XPD and XPA proteins limit L1 retrotransposition. (A) Schematic of the L1-retrotransposition assay. The L1 vector is a pCEP4 episomal vector carrying a hygromycin resistance (hygro^R^) gene, for the selection of transfected cells, and a full length L1 element tagged at the 3′ end with a retrotransposition cassette. The retrotransposition cassette consists of a reporter gene such as blasticidin resistance (blast^R^) or a neomycin resistance (neo^R^) gene interrupted with an intron. The reporter gene is in the reverse orientation in comparison to the L1 element and its transcription is driven by its own promoter, whereas the intron is in the sense orientation (direction of transcription is indicated by arrows). The splice donor and acceptor of the intron are indicated as SD and SA. The resistance gene becomes functional after L1 transcription, splicing, and TPRT of a new L1 copy into the genome. Therefore, only when retrotransposition of the cassette occurs can cells grow under selection conditions (blast^R^ or neo^R^ colonies). (B) Schematic of the protocol of the L1-retrotransposition assay used with the hygro^R^ pCEP4 episomal vectors. The day after transfection, cells containing plasmid are selected with hygromycin for 3 days. Then, selection medium is removed and replaced by growth medium for recovery. Ten days post-transfection, cells were reseeded in six-well plates at serial dilution, from 10^6^ to 10^3^ cells, in triplicate and grown under the appropriate selection. Two weeks later, cells were fixed and stained in crystal violet solution and the number of blast^R^ or neo^R^ colonies in each well is counted. (C) Relative retrotransposition rates of a blast^R^-tagged L1 reporter element (TAM102/L1.3) were determined in an XPA-deficient cell line (XPA-) and a commercially available stably complemented XPA-deficient cell line (XPA+). The results were normalized relative to XPA+, which was arbitrarily set to 1.0. L1 retrotransposition assay was performed three times independently. Bars represent the average and SEM from the three independent experiments. Statistical significance is indicated by * *P*-value = 1 × 10^−15^ (two-tailed, two-sample *t*-test). (D) Representative example of blast^R^ colony formation resulting from L1 retrotransposition assay performed in XPA+ and XPA- cells. The L1 retrotransposition rate for each cell line is indicated below. (E) Relative number of blast^R^ colonies resulting from L1 retrotransposition assay performed in XPD+ and XPD- cell lines. The results were normalized relative to the XPD+, which was arbitrarily set to 1.0. L1 retrotransposition assay was performed three times independently. Bars represent the average with SEM from the three independent experiments. Statistical significance is indicated by * *P*-value = 6.5 × 10^−6^ (two-tailed, two-sample *t*-test). (F) Representative example of blast^R^ colony formation resulting from L1 retrotransposition assay performed in XPD+ and XPD- cells. The L1 retrotransposition rate for each cell line is indicated below.

To determine whether the NER pathway controls L1 retrotransposition and is responsible for the role of the ERCC1-XPF complex in preventing L1 mobility, we investigated the effect of the deficiency of XPA and XPD, two crucial factors of the NER pathway, on L1 retrotransposition. XPA recruits ERCC1-XPF to the site of damage by directly binding the complex (Figure 1B) (Volker *et al.* 2001; Saijo *et al.* 2004). The helicase activity of the XPD protein forms part of the core structure of the transcriptional/DNA repair complex TFIIH, which opens the chromatin around the lesion during the NER process (Figure 1B and Coin *et al.* 1998). We performed L1 retrotransposition assays with marked L1 elements (Moran *et al.* 1996) in XPA- and XPD-deficient cell lines, and their stably complemented partners, XPA+ and XPD+ cells. We transfected the cells with the episomal pCEP4 vector carrying a L1.3 element (TAM102/L1.3) tagged at its 3′ end with the blasticidin (*mblast*) retrotransposition cassette (Morrish *et al.* 2002). The principle of the assay (Figures 2, A and B) is based on the introduction of an intron into the reporter gene in a manner that the reporter gene becomes functional after transcription of the L1 element, splicing of L1 mRNA, and target-primed reverse transcription. Thus, the formation of blasticidin-resistant colonies, due to the expression of the reporter gene, indicates that retrotransposition had occurred. The results of the L1 retrotransposition assays in XPA-, XPA+, XPD-, and XPD+ cells showed that stable complementation of NER-deficiency greatly decreased the number of blast^R^ colonies (Figure 2, C–F). These results are not due to differences in L1 expression in the cells, because the quantification of L1 mRNA by RT-qPCR showed equal amounts of RNA in all the cells, whether deficient or proficient for the NER pathway (Figure S1). In addition, immunoblot analysis verified that XPA and XPD protein expression in the complemented XPA- and XPD-deficient cell lines was restored (Figure S2A). A UV sensitivity assay (Levy *et al.* 1995) validated that the repair of UV-caused damage is not efficient in XPA- and XPD-deficient cells (low cell survival at high UV dose exposure), and becomes efficient again in their complemented versions (Figure S2, B and C). The increase in L1 retrotransposition in NER-deficient cells does not seem to be explained by the growth rate of the different cell lines, because the cell cycle analysis by FACS revealed a slightly longer cycle for NER-deficient cells, as a few more cells accumulated in G2 phase (Figure S3). A longer cell cycle does not seem to be a significant factor of a retrotransposition rate increase in our studies, because previous publications have shown that, although transiting the cell cycle does not seem to be required for retrotransposition (Kubo *et al.* 2006), any arrest in the cell cycle seemd to lead to a decrease in L1 retrotransposition (Kubo *et al.* 2006; Shi *et al.* 2007; Xie *et al.* 2013).

As a control, we tested for the possibility that NER-deficiency would promote the retrotransposition of L1 elements defective in the endonuclease (EN) as has been seen in other DNA repair defects (Morrish *et al.* 2002). No blast^R^ colonies were observed performing retrotransposition assays in NER-deficient cells, using L1 constructs individually defective in either endonuclease or reverse transcriptase [TAMH230A102/L1.3, EN(-) or TAMD702A/L1.3, RT(-)] (Figure S4). In parallel, to rule out any bias engendered by various transfection efficiency, growth rates, and L1-caused toxicity, we verified that L1 expression did not alter colony formation in a manner that could cause retrotransposition rate differences in any of the cells used in the experiment (Figure S5).

### The GGR DNA lesion binding protein, XPC, also contributes to the limitation of L1 retrotransposition

NER can recognize and repair a wide variety of lesions that are not related in their chemical structures, such as bulky chemical DNA adducts and UV-induced lesions (Gillet and Schärer 2006). Some reports suggest that it is the helical distortion that attracts the NER repair proteins (Clement *et al.* 2010; Naegeli and Sugasawa 2011). Whether the DNA lesion occurs in a transcriptionally active or inactive region in the genome will influence the NER sensor used for the repair. Because L1 insertion occurs throughout the genome (Deininger and Batzer 2002; Morrish *et al.* 2002) and not just in transcribing genes, we evaluated the GGR DNA lesion-binding protein XPC for its ability to limit L1 retrotransposition. We monitored L1 retrotransposition events in XPC-deficient cells and in transiently complemented (XPC+) versions of the same cells.

**Figure 3:**
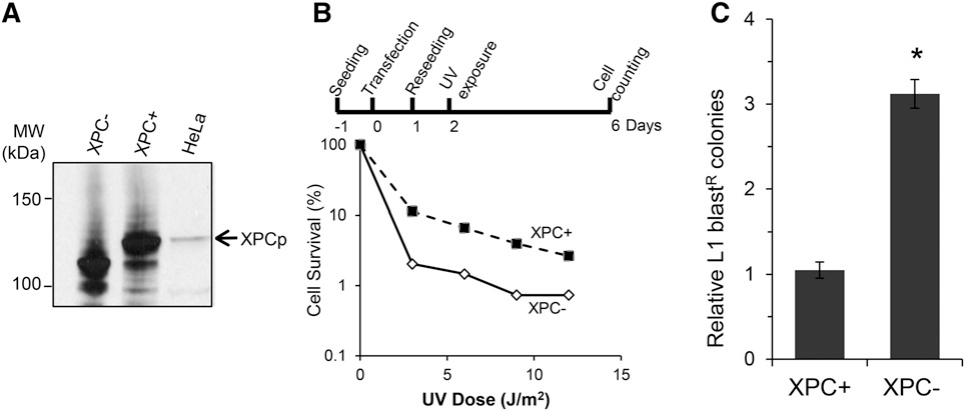
XPC limits L1 retrotransposition. (A) Expression of XPC protein (XPCp) in HeLa cells and in XPC-deficient cells, transfected with either wild-type XPC (pCMV6-XPC) (XPC+) or mutant XPC (pCMV6-XPCΔ) (XPC-) expression vector was analyzed by immunoblotting 24 hr post-transfection. The extra bands observed in XPC+ and XPC- lanes can be the truncated form of XPC protein, present in the XPC-deficient cell line and caused by two deletions in the XPC gene sequence, generating a frameshift and a truncated protein (Li *et al.* 1993). The truncated protein might be stabilized by the overexpression of the wild-type or mutant XPC protein. (B) Determination of the nucleotide excision repair competency of the transient complementation of the XPC-deficient cells with a wild-type XPC (pCMV6-XPC)- or mutant XPC (pCMV6-XPCΔ)-expressing vector by using a UV sensitivity assay. Top panel shows the schematic of the UV sensitivity assay (details in *Materials and Methods*). XPC- cells and its transiently complemented version, XPC+ cells, were exposed to 0, 3, 6, 9, or 12 J/m^2^ UVC dose. Cell survival was determined 4 days post UV exposure. Data represent the logarithm of the percentage of cell survival plotted against the UV dose. The assay was performed at least three times independently for each condition, a representative is shown. (C) Relative number of blast^R^ colonies resulting from the L1 retrotransposition assay performed in the XPC-deficient cell line and its transiently complemented version. XPC- cells were cotransfected with XPC cDNA (XPC+) or control (XPC-) vectors and the tagged L1 vector (TAM102/L1.3). The L1 retrotransposition assay was performed as described for Figure 2. The results were normalized to the XPC+ result, which was arbitrarily set to 1.0. Data correspond to the relative number of blast^R^ colonies determined in each condition. L1 retrotransposition assays have been performed independently at least three times. Data represent the average with SEM. Statistical significance is indicated by *P-value: 0.0001 (two-tailed, two-sample *t*-test). MW, molecular weight.

Monitoring XPC protein (XPCp) expression level and repair efficiency of the UV-induced DNA damage, we determined that the transient transfection of XPC-deficient cells resulted in a partial complementation of the deficiency (Figure 3, A and B). In fact, the cell survival rate was under 10% for moderate UV dose (6 J/m^2^), while the cell survival rates of stably complemented XPA+ and XPD+ cell lines and HeLa cells were about 50% for the same UV dose (Figure S2, B and C). However the cell survival of transiently complemented XPC- cells (XPC+) using a vector expressing XPC cDNA (pCMV6-XPC), which expresses a large amount of XPCp (Figure 3A), was significantly higher than the cell survival of the XPC-deficient cells (XPC-) transfected with the vector control (pCMV6-XPCΔ) (*P* < 0.0001, Chi-square test for trend) (Figure 3B). Showing the same trend as in XPA- and XPD-deficient cells, L1 retrotransposition increased threefold in the XPC-deficient cells (XPC-) in comparison to the partially complemented cells (XPC+) (Figure 3C). These results indicate that the lesion binding protein in the GGR pathway, XPCp, is likely involved in the regulation of L1 retrotransposition.

### The deficiency in NER proteins does not have an impact on the size of *de novo* L1 inserts, but *de novo* L1 inserts present abnormally large target site duplications in NER-deficient cell lines

Because complementation of NER-deficiency leads to a decrease in the apparent retrotransposition rate, suggesting that NER proteins inhibit retrotransposition events, we wished to investigate whether mutations in NER proteins impact the characteristics of *de novo* L1 inserts. We extracted the genomic DNA of XPC-, XPA-, and XPD-deficient cells and in the stably complemented partner, XPD+, expressing the marker of retrotransposition. We then sequenced *de novo* inserts and the flanking regions to evaluate the features of the inserts, such as the length of the insert, the length of the polyA tail, the size of the target site duplication (TSD), and the sequence of the L1 endonuclease cleavage site. The principle of the method is presented in Figure S6A. The previously published method (Morrish *et al.* 2002; Yuanxin *et al.* 2003; El-Sawy *et al.* 2005; Gasior *et al.* 2007) was slightly modified to facilitate analysis of the 3′ flanking sequences of the L1 elements recovered as plasmids (details in *Materials and Methods*). Because the stably complemented cell lines were resistant to neomycin, we could not use the previously described synL1_neo vector (El-Sawy *et al.* 2005). As a result, we designed a new rescue vector, pBS-L1PA1_CH__blast, using a blasticidin retrotransposition cassette (Figure S6B). Except for the insert size that depends on the length of the reporter cassette, the characteristics of *de novo* L1 inserts obtained with the new L1PA1_CH__blast rescue vector in HeLa cells were similar to those previously published in (Gilbert *et al.* 2002, 2005; Symer *et al.* 2002) (Table S1 and Table S2).

We recovered 29 *de novo* L1 inserts in XPD-deficient cells (XPD-), 9 in XPA-deficient cells (XPA-), and 26 in XPC-deficient cells (XPC-) using the synL1_neo vector and 17 in stably complemented XPD-deficient cells (XPD+) using the L1PA1_CH__blast rescue vector (Table 1, Table 2, Table 3, and Table 4). We particularly focused on recovering L1 inserts in the XPD-deficient cell lines to allow a statistically significant analysis. The recovered inserts were dispersed randomly throughout the genome with no noticeable preference for a specific site or orientation on chromosomes. No full-length *de novo* L1 insertions were recovered in NER-deficient cells or in the stably complemented cell line XPD+. The inserts were all 5′ truncated and the size of the inserts varied from 1.8 to 2.8 kb in the complemented cells and from 2.3 to 4.3 kb in NER-deficient cells (the minimal recoverable size of the insert is 1.8 kb using L1PA1_CH__blast rescue vector for the assay or 2.3 kb using synL1_neo vector as the presence of the selection marker, blasticidin, or neomycin, respectively, and the bacterial origin of replication at the 3′ end of the L1 construct are required for recovery). In comparison with data obtained in HeLa cells, we detected no significant difference in the median length of L1 inserts in NER-deficient and proficient cells lines (*P*-value Mann–Whitney *U*-test > 0.05, Figure S7, A and B). Therefore, NER proteins do not seem to influence the size of the *de novo* L1 inserts.

**Table 1:** Characteristics of *de novo* L1 inserts recovered in XPD-deficient cells

Clone #	Insert Size	Chromosome	Orientation	Position (5′–3′ End)	TSD Length	TSD Verification	pA Length	Cleavage Site
DM.1	2663	Chr5	+	137,495,110–137,495,108	2	S	41 nt	TCTT/a
DM.2	2883	Chr18	—	57,193,322–57,193,327	5	S	55 nt	TTTA/a
DM.3	3147	Chr12	+	112,775,935–112,775,928	7	S	45 nt	CTTT/a
DM.4	2780	Chr7	—	61,968,906–61,968,919	13	S	81 nt	TTTC/a
DM.5	2827	Chr12	—	98,026,422–98,026,438	16	S	30 nt	TTTT/a
DM.6	3929	Chr8	+	128,728,905–128,728,851	54	S	58 nt	TCTT/a
DM.7	3989	Chr17	—	13,596,243–13,596,460	217	S	83 nt	TTTT/a
DM.8	2804	Chr17	—	50,628,899–50,629,306	407	S/P	—	CTAG/a
DM.9	3626	Chr1	—	121,484,567–121,484,978	411	S	73 nt	TTCT/g
DM.10	3686	Chr3	—	174,149,054–174,149,550	496	S/P	84 nt	TCTT/a
DM.11	2550	Chr7	+	144,760,424–144,759,911	513	S/P	34 nt	TTTT/g
DM.12	3008	Chr3	+	29,453,614–29,453,069	545	S/P	25 nt	TTTT/a
DM.13	3167	Chr11	+	105,190,706–105,190,152	554	S/P	46 nt	ACTT/g
DM.14	2559	ChrX	—	10,708,604–10,709,170	571	S/P	77 nt	TTCT/a
DM.15	3719	Chr15	+	30,302,496–30,301,796	700	S/P	—	CCCA/g
DM.16	3103	Chr8	—	58,341,572–58,342,341	769	S/P	97 nt	TTTC/a
DM.17	3411	Chr14	—	77,523,239–77,524,010	771	S/P	69 nt	TTTT/a
DM.18	3610	Chr9	+	5,534,460–5,533,597	863		37 nt	TTTT/a
DM.19	2928	ChrX	+	33,775,626–33,774,740	886	S/P	33 nt	TTTA/g
DM.20	2582	Chr7	—	130,375,857–130,376,914	1057	S/P	—	TGGT/c
DM.21	2380	Chr9	+	40,823,585–40,822,515	1070	S/P	56 nt	CTTT/a
DM.22	2894	ChrX	+	122,778,988–122,777,801	1187		39 nt	TTTC/a
DM.23	2536	Chr5	+	64,034,619–64,033,086	1533	P	19 nt	TTTT/c
DM.24	3917	Chr11	+	14,727,287–14,725,642	1645		57 nt	TTTC/a
DM.25	3857	Chr1	—	120,251,234–120,249,499	1735	S/P	65 nt	GTTT/g
DM.26	3991	Chr3	+	29,455,524–29,453,069	2455	P	22 nt	TTTT/a
DM.27	3940	Chr15	—	28,869,801–28,873,325	3361	P	87 nt	TATT/a
DM.28	3996	Chr13	—	44,857,642–44,861,667	4025	P	57 nt	TTTG/a
DM.29	2655	Chr3	+	29,458,420–29,453,069	5896		30 nt	TTTT/a

TSD, target site duplication; Chr, chromosome; S, sequence; P, PCR.

**Table 2:** Characteristics of *de novo* L1 inserts recovered in (XPD+) complemented XPD-deficient cells

Clone #	Insert Size	Chromosome	Orientation	Position (5′–3′ End)	TSD Length	Deletion Length	pA Length	Cleavage Site
DP.1	1956	Chr3	+	166,704,010–166,704,008	3		31 nt	TTTT/a
DP.2	1989	Chr8	+	121,836,950–121,836,947	4		31 nt	TCTT/a
DP.3	2318	Chr20	+	52,652,085–52,652,079	7		38 nt	TTTA/a
DP.4	2270	Chr3	—	156,453,738–156,453,742	4		28 nt	CTTT/a
DP.5	2269	Chr7	+	90,201,740–90,201,726	15		45 nt	TTTC/a
DP.6	2038	Chr16	—	53,268,517–53,268,532	16		33 nt	TTTT/a
DP.7	1866	Chr8	+	30,763,655–30,763,645	11		46 nt	TTAT/a
DP.8	2013	Chr2	—	24,215,408–24,216,927	1520		35 nt	TTTT/a
DP.9	1977	Chr17	+	35,458,775–35,457,164	1612		40 nt	TCTT/a
DP.10	2704	Chr12	—	18,901,143–18,901,142	—	—	52 nt	TCTT/a
DP.11	1866	Chr15	—	87,439,350–87,439,347		2	52 nt	TTTA/a
DP.12	2791	Chr6	+	99,948,429–99,948,435		5	19 nt	TTTT/a
DP.13	1900	Chr14	—	51,873,941–51,873,933		7	25 nt	TTTG/a
DP.14	2062	Chr1	—	94,735,344–94,735,334		9	34 nt	TCTT/a
DP.15	2380	Chr4	—	30,539,318–30,539,303		14	25 nt	TTTT/a
DP.16	2002	Chr2	—	59,684,354–59,684,338		15	110 nt	TTCT/a
DP.17	2741	Chr6	—	248,880–248,410		469	55 nt	TTTT/a

TSD, target site duplication; Chr, chromosome.

**Table 3:** Characteristics of *de novo* L1 inserts recovered in XPC-deficient cells

Clone #	Insert Size	Chromosome	Orientation	Position (5′–3′ End)	TSD Length	Deletion Length	pA Length	Cleavage Site
CM.1	2491	Chr15	—	52,326,493–52,326,495	2		25 nt	TTTT/a
CM.2	2736	Chr4	+	58,109,300–58,109,280	20		29 nt	TTTA/a
CM.3	2421	Chr4	—	81,232,879–81,233,154	275		—	ATTT/a
CM.4	3167	Chr5	+	41,311,414–41,311,137	277		60 nt	TCTT/g
CM.5	2886	Chr15	+	33,641,486–33,640,983	503		15 nt	TTTT/a
CM.6	2661	Chr8	+	23,490,649–23,489,121	516		18 nt	TGAA/a
CM.7	2946	Chr2	+	182,583,931–182,583,379	552		—	AATA/a
CM.8	2886	Chr6	—	85,235,896–85,236,510	614		—	AATT/t
CM.9	2567	Chr5	—	65,162,148–65,162,763	615		—	AATG/a
CM.10	2609	Chr16	—	9,557,849–9,558,491	642		10 nt	TTTT/a
CM.11	3386	Chr8	+	26,232,474–26,231,790	684		25 nt	TCTT/a
CM.12	2677	Chr9	+	66,811,576–66,810,877	699		15 nt	TTTT/g
CM.13	3293	Chr17	+	49,728,154–49,727,312	842		6 nt	GATT/g
CM.14	3112	Chr11	—	114,392,300–114,393,187	887		23 nt	TCTT/a
CM.15	2894	Chr3	+	34,830,281–34,829,315	966		—	AGTT/c
CM.16	2604	Chr1	—	211,947,488–211,948,623	1135		—	CTGC/a
CM.17	2684	Chr8	+	127,633,155–127,631,920	1235		84 nt	TTCT/a
CM.18	3044	Chr3	+	5,007,476–5,005,443	2033		—	ATTT/t
CM.19	3096	Chr17	+	48,833,262–48,831,219	2043		21 nt	TTTC/a
CM.20	2718	Chr20	—	12,381,555–12,384,581	3026		—	ACCT/g
CM.21	3035	Chr18	+	20,321,307–20,317,622	3685		—	TAAG/c
CM.22	2890	Chr2	—	137,563,056–137,567,601	4545		31 nt	TTTT/a
CM.23	4006	Chr19	—	27,732,520–27,739,285	6765		38 nt	TTTT/g
CM.24	2700	Chr18	—	22,016,206–22,016,201		5	21 nt	CTTT/a
CM.25	4306	Chr14	—	67,575,620–67,575,614		6	20 nt	TTAA/g
CM.26	4192	Chr9	+	115,010,933–115,010,942		9	13 nt	TTTT/a

TSD, target site duplication; Chr, chromosome.

**Table 4:** Characteristics of *de novo* L1 inserts recovered in XPA-deficient cells

Clone #	Insert Size	Chromosome	Orientation	Position (5′–3′ End)	TSD Length	Deletion Length	pA Length	Cleavage Site
AM.1	2490	Chr2	—	36,487,725–36,487,738	13		35 nt	TTTC/a
AM.2	4230	Chr3	—	47,259,213–47,259,253	40		79 nt	TTTC/a
AM.3	2678	Chr11	+	85,736,502–85,736,455	47		42 nt	TCTT/a
AM.4	3409	Chr15	—	28,728,325–28,728,567	242		25 nt	TTTC/a
AM.5	2265	Chr9	—	135,357,719–135,359,341	1622		5 nt	TTTT/g
AM.6	2712	Chr2	+	89,248,581–89,246,458	2123		—	ACTG/a
AM.7	2756	Chr12	—	19,866,410–19,866,397		13	9 nt	TTTT/a
AM.8	2306	Chr3	+	36,006,942–36,010,967		4025	50 nt	TGTT/a

TSD, target site duplication; Chr, chromosome.

The majority of *de novo* L1 inserts occurred at typical L1 endonuclease cleavage sites (Table 1, Table 2, Table 3, and Table 4 and Morrish *et al.* 2002). However, 1 out of the 9 (11%) recovered inserts (clone #AM7, Table 4) in XPA-deficient cells, 9 out of 26 (35%) in XPC- cells (clones #CM3, #CM7, #CM8, #CM9, #CM15, #CM16, #CM18, #CM20, and #CM21, Table 3), and 3 out of 28 (11%) in XPD- cells (clones #DM8, #DM15, and #DM20, Table 1) occurred at an atypical cleavage site and also did not contain the polyA tail at the 3′ end of the L1 sequence, a hallmark of an L1 insertion event. Inserts similar to these have been previously described as the result of endonuclease-independent insertion events in DNA repair-deficient cells (Morrish *et al.* 2002). However, in the present study, the frequency of these events was significantly higher in XPC-deficient cells than in the other NER-deficient cells and HeLa cells (Gilbert *et al.* 2005), and we did not observe any retrotransposition events in NER-deficient cells transfected with a L1 construct defective in the endonuclease activity of ORF2p (Figure S4). Therefore, endonuclease-independent insertion of L1 in NER-deficient cells does not seem to explain these events, and these atypical inserts may be the results of recombination events between L1 and genomic sequences occurring at the insertion site during the insertion process.

Genomic deletions at the L1 insertion site were observed in 3 out of 26 (12%) in XPC-deficient cells (clones #CM24 to #CM26, Table 3) and 2 out of 9 (22%) in XPA-deficient cells (clones #AM8 and #AM9, Table 4). These observations are similar in frequency to small deletions found from an analysis of polymorphic genomic L1 inserts (Konkel *et al.* 2007), although our deletions were slightly longer in general. A significantly higher rate of deletions was observed in the complemented XPD+ cells (8 out of 17, 47%). For nearly half of these events, deletions were generated by homologous recombination between the sequences of the L1 element in the genome at the insertion site and the elongating *de novo* L1 (see TPRT process, Figure 1A). This type of event has been previously reported and described in HeLa cells, although not at this high rate (Gilbert *et al.* 2005).

**Figure 4:**
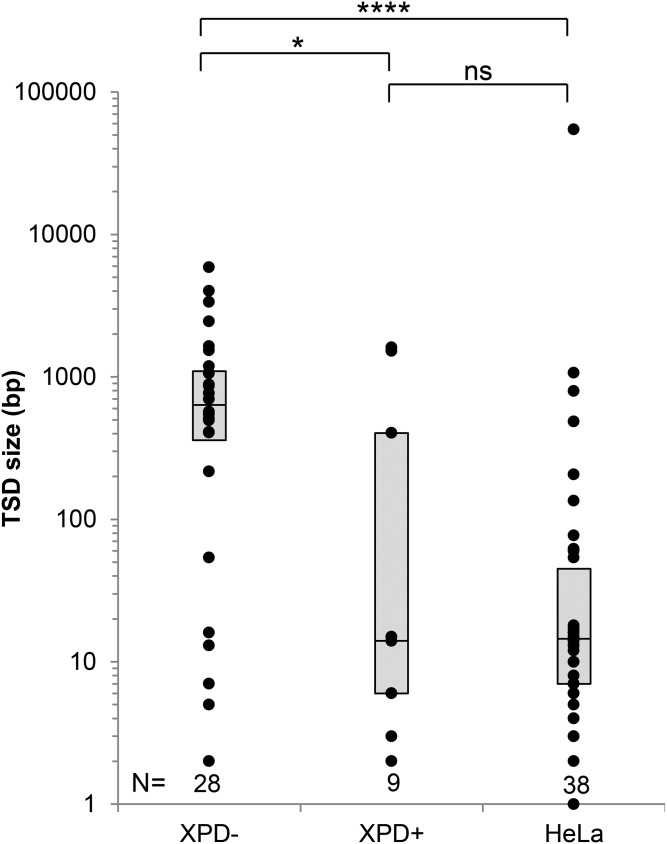
Recovered *de novo* L1 inserts in NER-deficient cells have large TSDs. Dot plot representation of the size of TSDs of *de novo* L1 inserts recovered from XPD-deficient cells (XPD-), stably complemented XPD-deficient cells (XPD+) and HeLa cells. The boxes represent the interquartile range (the range between the first and third quartiles) of the distribution of TSD size for each cell line. The line in the middle of the box represents the median. The count (*n*) of recovered inserts for each cell line is indicated above the name of the cell line. Statistical significance is indicated by *P-value = 0.032, ****P-value = 3.13 × 10^−7^ (*P*-value = 0.38) (Mann–Whitney *U* test). NER, nucleotide excision repair; TSD, target site duplication.

One area in which NER-deficient L1 inserts differed from those in NER-proficient cells is the size of the target size duplication. In NER-deficient cells, TSDs ranged from 5 bp to 5.3 kb in size with a median length of 1495 bp, while in XPD+ cells, TSDs ranged from 2 bp to 1.6 kb in length with a median of 14 bp (Table 1, Table 2, Table 3, and Table 4). TSD length in NER-deficient cells was significantly larger than those observed in either cells stably complemented to correct the NER deficiency or in NER-proficient HeLa cells (*P*-value Mann–Whitney *U*-test < 0.05, Figure 4). Previous studies have also reported that the typical size of a TSD from L1 inserts recovered from normal culture cells and from an L1 insert naturally occurring in the genome is usually 5–30 bp (Morrish *et al.* 2002; Symer *et al.* 2002; Gilbert *et al.* 2005). Events generating large TSDs (> 100 bp) have been observed in HeLa cells, but the frequency of these events never exceeded 10% of the total recovered events (Table S1 and Table S2) (Gilbert *et al.* 2002, 2005; Symer *et al.* 2002). In the NER-deficient cell lines tested in the present study, 4 out of 7 (57%) *de novo* inserts on XPA- cells, 21 out of 23 (91.3%) in XPC- cells, and 22 out of 28 (78.6%) in XPD- cells presented TSDs over 100 bp in length (Figure 4 and Figure S8) and of these, 18 (38%) had TSDs of over 1 kb in length. Only three out of nine (33%) events presented TSDs over 100 bp in the stably complemented cells (XPD+), a difference that is statistically significant (*P*-value Fisher’s exact test < 0.05). It seems possible that the slightly elevated levels of longer TSDs in the complemented cells relative to previous studies in HeLa, for instance, may be due to the complementation not necessarily bringing the NER pathway to maximum efficiency. Therefore, our findings seem to imply that the expression of NER proteins is required to prevent large genomic rearrangements at the L1 insertion site.

## Discussion

L1 elements have a tremendous capacity to cause damage to the human genome, potentially causing disease, cell death, and aging (Belgnaoui *et al.* 2006; Gasior *et al.* 2006; Wallace *et al.* 2010; Belancio *et al.* 2014). Because L1 retrotransposition is a threat to genome integrity, it is not surprising that numerous cellular factors control the mobility of these elements at different levels. For example, L1 expression is maintained at a low level by methylation (Alves *et al.* 1996), premature polyadenylation, and alternative RNA splicing (Belancio *et al.* 2008b). In addition, at a post-transcriptional level, members of the human cytidine deaminase APOBEC3 family, involved in the response against viral infection, inhibit the activity of L1 retrotransposons (Bogerd *et al.* 2006; Kinomoto *et al.* 2007; Lovsin and Peterlin 2009; Richardson *et al.* 2014).

Our data demonstrate that the entire global NER pathway represents an additional mechanism regulating L1 activity. The ERCC1-XPF complex, previously identified as an L1 regulator, is involved in many DNA repair pathways and is more essential for life than the other NER components. All three NER proteins tested in this study (XPAp, XPDp, and XPCp, Figure 2 and Figure 3) show a similar ability to suppress L1 retrotransposition as previously reported for the ERCC1-XPF complex (Gasior *et al.* 2008). This suppression seems likely to be the result of an altered ability to complete the retrotransposition process successfully, rather than an ability to make longer inserts in NER deficiency, given that we detected no significant differences in insert length (Figure S7).

It seems likely that the NER pathway is primarily responsible for the inhibition of L1 retrotransposition by ERCC1-XPF, as the magnitude of the effect of XPA, XPD, and ERCC1 deficiencies on L1 retrotransposition were very similar. However, this does not rule out the possibility that some of the other sensors and pathways that recruit ERCC1-XPF to DNA lesions may also be involved, to a lesser extent, in the control of L1 insertion. In particular, TCR, the other NER subpathway, may also contribute to the sensing of L1 insertion events. However, because TCR only protects the portion of the genome that is being actively transcribed in that specific cell type (Mellon *et al.* 1987), the TCR subpathway could only have a more limited effect on L1 insertion rates. Because the TCR pathway is strand-specific, its influence would primarily be limited to influencing the orientation preference of the insertion within genes rather than on the global rate of retrotransposition events.

Previously, the Garfinkel laboratory reported that Ssl2 and Rad3, two DNA helicases of the TFIIH complex and homologs of human XPB and XPD proteins, respectively, can inhibit Ty1 retrotransposition in *Saccharomyces cerevisiae* (Lee *et al.* 1998). Ssl2 and Rad3 helicases are proposed to induce the degradation of Ty1 cDNA in the cytoplasm by destabilizing its structure. However, Ty1 and L1 elements have very different integration mechanisms. The former is a virus-like retrotransposon, similar to retroviruses, and its reverse transcription occurs in a pseudoviral particle in the cytoplasm. The cDNA is imported into the nucleus and is integrated in the genome. In contrast, L1 mRNA is reverse transcribed inside the nucleus (TPRT), directly at the site of the insertion of the new copy. Therefore, it is not expected that these two kinds of elements would induce a similar cellular response. Intriguingly, in both cases, it seems likely that the NER components are involved in limiting the insertion by limiting the synthesis/stability of the cDNA intermediate.

**Figure 5:**
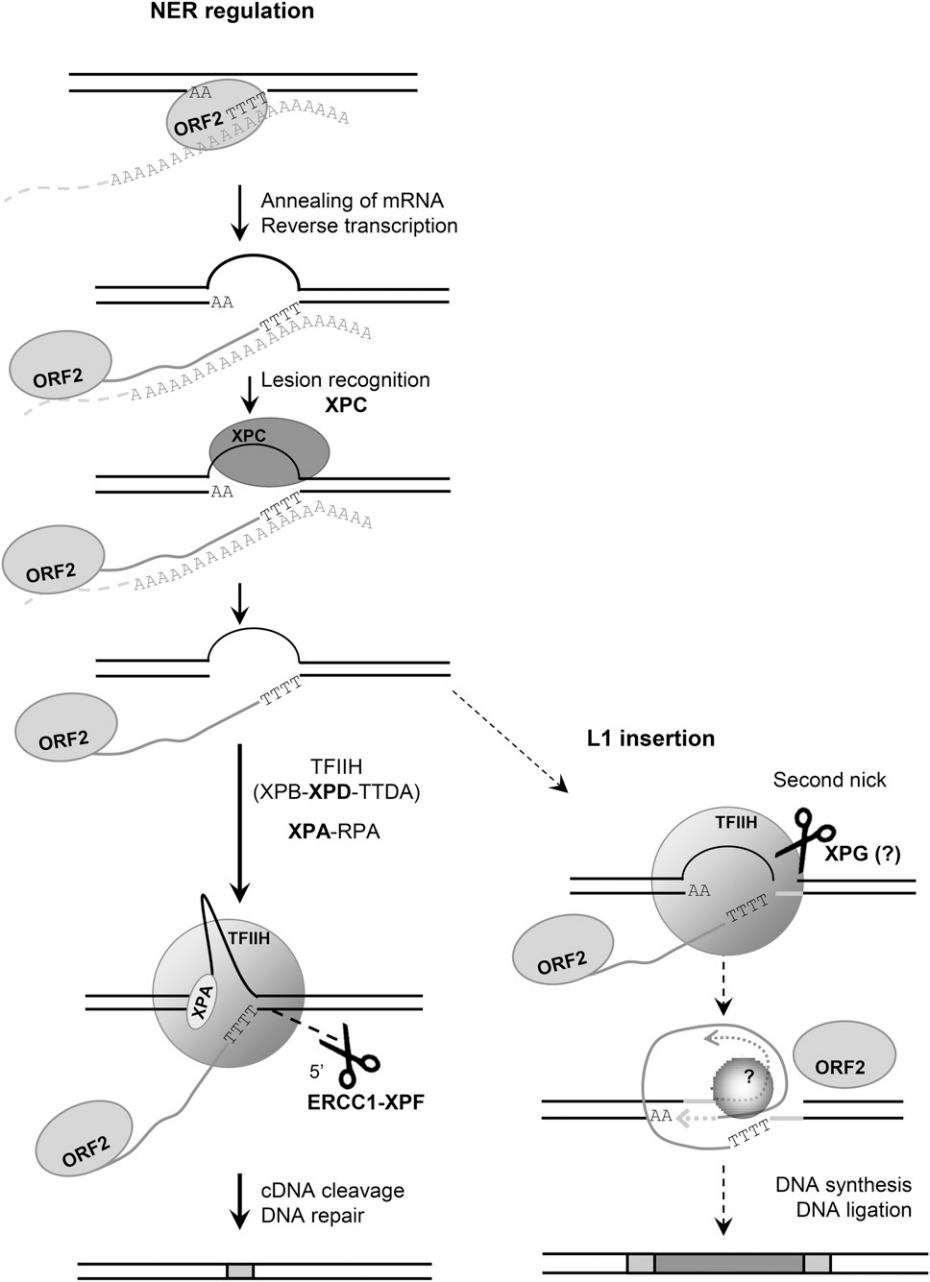
Model of limitation of damage caused by L1 TPRT via the GGR-NER pathway. The 3′ flap DNA structure is generated by L1 elongating cDNA during TPRT process. The XPC complex recognizes the structure and recruits the other proteins of the NER pathway to the L1 insertion site. ERCC1-XPF endonuclease cleaves the elongating L1 cDNA, inhibiting the insertion of a new L1 element in the genome and leading to the restoration of the original DNA sequence. To a lesser extent, the NER pathway seems to be involved in generating a second nick during the L1 insertion process, in close proximity to the first cleavage generated by the L1 endonuclease. Therefore, in WT cells, the *de novo* insert is flanked by small (< 100 bp) TSDs, whereas in NER-deficient cells, a more distal unrelated nick may be used to complete the retroelement insertion resulting in large TSDs. cDNA, complementary DNA; GGR, global genome repair; mRNA, messenger RNA; NER, nucleotide excision repair; RNAPII, RNA Polymerase II; TCR, transcription coupled repair; TPRT, target-primed reverse transcription; TSD, target site duplication.

In addition to the role of the NER factors in limiting the insertion of a new copy of L1, our L1 insert recovery data suggest that the NER pathway may also be involved in the less well-defined last step of the L1 TPRT process, as a high number of extremely large TSDs was observed in NER-deficient cell lines. These results suggest that the NER factors present at the site of L1 insertion may influence the formation or selection of the second nick necessary for the completion of the TPRT process at a location close to the cleavage site of the L1 endonuclease (Figure 5). Although speculative, in NER-deficient cells, the location of the second nick is highly variable and might depend on a more random event that can occur at a location very far away from the primary cleavage site, thereby generating extremely large TSDs. The distant location of the second nick can be the result of the direct influence of the chromosomal region on the L1 insertion process, such as exposed DNA ends generated during replication or DNA repair processes, serving as primers for the synthesis of the second L1 cDNA strand. An alternative possibility is that the predicted cDNA flap intermediate may persist in ERCC1-XPF-deficient cells, giving the insertion process a longer time to reach a naturally occurring nick at a more distant location through the strand displacement activities of helicases recruited to the site of L1 insertion by DNA repair machinery.

In addition to the long TSDs in these L1 insertions, the only other unusual feature is an unusually high number of events that do not look like they went through the normal TPRT process on the polyA tail. Although most of the inserts have the typically long A tails expected of *de novo* L1 insertions, these “tailless” inserts seem to indicate some other undefined aberrant processes during the insertion.

The extremely large TSDs found in NER-deficient L1 insertions fall within the size range of genomic copy number variations (Eichler 2001). This large size makes them potentially more deleterious as insertions. However, it also has the consequence of duplicating regions that may have functional significance, such as exons. Thus, they represent a unique source of this type of genomic duplication. However, it is also worth considering that large segments of homology near one another also have a propensity to recombine with one another (Stankiewicz and Lupski 2002). This would be similar to the formation of solo LTRs following retroviral insertion (Belshaw *et al.* 2007). Thus, this type of event might be expected to have a shortened life span in the genome and some L1 insertions may be eliminated by recombination between their long TSDs. Our data suggest that individuals with defects in NER, such as XP patients, are likely to be subject to higher levels of DNA damage caused by L1 elements. Other studies reported that NER capacity varies between individuals (Spitz *et al.* 2001; Tomescu *et al.* 2001; Hou *et al.* 2002) in response to different stimuli such as circadian rhythm (Gaddameedhi *et al.* 2011), or are specifically repressed in some cancers (Xu *et al.* 2011). Under those conditions and in combination with a deregulation of L1 expression, NER deficiency may increase the potential impact of L1 retrotransposition on the human genome, favoring somatic genetic instability and the generation of age-related diseases, such as cancer.

## 
